# Monitoring tissue oxygenation by near infrared spectroscopy (NIRS): background and current applications

**DOI:** 10.1007/s10877-012-9348-y

**Published:** 2012-03-31

**Authors:** T. W. L. Scheeren, P. Schober, L. A. Schwarte

**Affiliations:** 1 Department of Anaesthesiology, University Medical Center Groningen, University of Groningen, Groningen, The Netherlands; 2 Department of Anaesthesiology, VU University Medical Center, Amsterdam, The Netherlands

**Keywords:** Monitoring, Tissue oxygenation, Near infrared spectroscopy, Cerebral oxygenation, Prehospital evaluation

## Abstract

Conventional cardiovascular monitoring may not detect tissue hypoxia, and conventional cardiovascular support aiming at global hemodynamics may not restore tissue oxygenation. NIRS offers non-invasive online monitoring of tissue oxygenation in a wide range of clinical scenarios. NIRS monitoring is commonly used to measure cerebral oxygenation (rSO_2_), e.g. during cardiac surgery. In this review, we will show that tissue hypoxia occurs frequently in the perioperative setting, particularly in cardiac surgery. Therefore, measuring and obtaining adequate tissue oxygenation may prevent (postoperative) complications and may thus be cost-effective. NIRS monitoring may also be used to detect tissue hypoxia in (prehospital) emergency settings, where it has prognostic significance and enables monitoring of therapeutic interventions, particularly in patients with trauma. However, optimal therapeutic agents and strategies for augmenting tissue oxygenation have yet to be determined.

## Introduction

Adequacy of tissue oxygenation is a prerequisite of aerobic metabolism. Thus, in many fields of medicine, therapy aims to maintain, restore, or optimize tissue oxygenation [1, 2]. However, it remains a challenge to clinically assess tissue oxygenation on a routine basis. Since occult regional ischemia, i.e., not detectable at the systemic level, is considered a major contributor to morbidity and mortality after major trauma and in other critically ill patients, methods are required to permit early detection of regional hypoxia/dysoxia and guide therapy to restore it [3]. On the other hand it is increasingly realized that optimizing tissue oxygenation does not follow a simple more-is-better doctrine, i.e., achieving supranormal goals of oxygenation may be detrimental as well. Thus therapy to optimize oxygenation should be directed to specific oxygenation goals, including regional oxygenation goals, and therefore monitoring becomes essential.

Systemic arterial and (mixed/central) venous oxygenation can be measured routinely with widely established techniques like pulse oxymetry, blood gas analysis, and venous fiber oxymetry (e.g., in pulmonary artery catheters). However, regional measurement of tissue oxygenation was not possible on a routine clinical basis until recently. Traditionally, tissue oxygenation had been measured merely by experimental tools, that were either invasive (e.g., Clark-type needle electrodes) or depend on toxic dyes (e.g., palladium phosphorescence), restricting their clinical use. Within the last decade promising techniques are introduced with the potential to routinely assess tissue oxygenation. The clinically most broadly spread technique is near infrared spectroscopy (NIRS). Beside a brief introduction to the technique, a brief review over the literature and own clinical experiences with this technique will be presented.

## Technical background

The technical background of NIRS has been presented in detail recently [4] and will therefore be presented here only briefly. Distinct biological molecules change their optical properties when binding to oxygen. From experience, one recalls that highly oxygenated blood appears red, whereas de-oxygenated blood appears dark, ranging from blue to black. This phenomenon is caused by the fact that oxygenated hemoglobin differs in parts of it’s absorption pattern from de-oxygenated hemoglobin, and thus in their apparent optical spectrum. These optical differences have been exploited and are now clinical standard application in pulse oxymetry, where usually two or three distinct wavelengths are used in combination with pulse plethysmography to measure the arterial hemoglobin oxygen saturation.

**Fig. 1:**
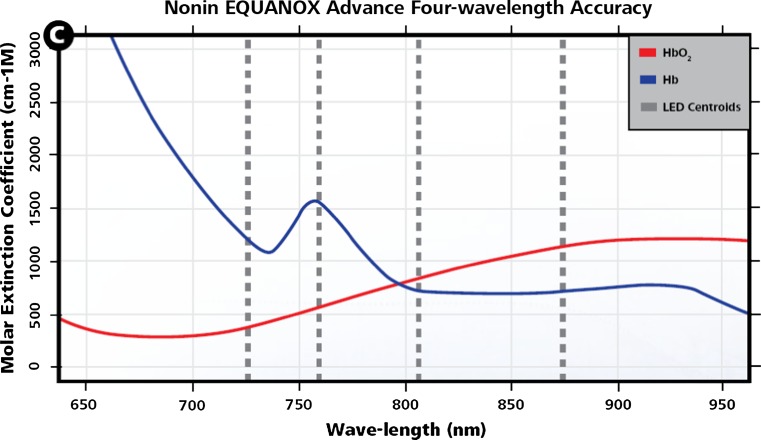
NIRS. Optical properties of oxy- and desoxyhemoglobin. From Nonin with kind permission

**Fig. 2:**
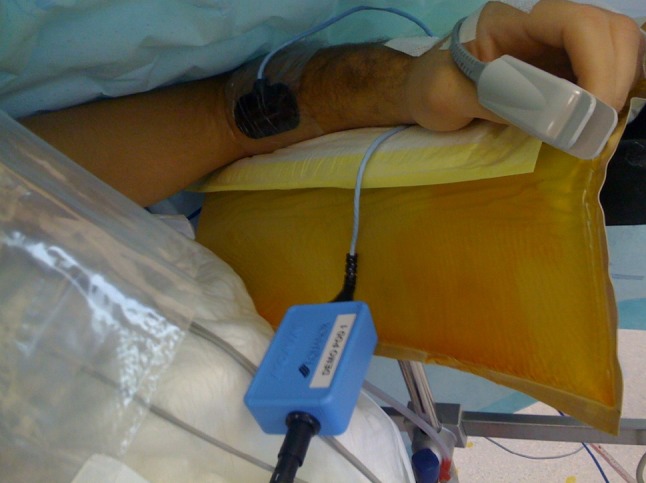
Probe positioning. Example of forearm probe positioning. Here additionally fixated with a transparent adhesive

Visible light penetrates tissue only short distances, since it is markedly attenuated by several tissue components, which absorb or scatter visible light. However, in the NIR spectrum (ranging from 700 to 1100 nm) photons are capable of deeper penetration of several centimeters or more. Contra-intuitively, NIR beams may also penetrate bones, which is prerequisite for trans-cranial cerebral oxymetry. Aside from the advantage of relatively deep penetration of several centimeters, the NIR spectral region is also characterized by typical differences in the spectrum of oxygenated and deoxygenated hemoglobin (Fig. 1). Exploiting these natural characteristics for regional oxymetry, a prototypical NIRS monitor functions as follows: A light source (e.g., a LED) generates NIR light, concerning the spectrum centered around characteristic wavelengths (examples are given in Fig. 1). The emitted beam is directed into the tissue of interest via a (usually cutaneously attached) probe. Whereas in some devices the NIR light is generated within the actual monitor module, and thereafter directed via glass fibers to the probe tip (InSpectra, Hutchinson Technology Inc., BioMeasurement Division, Hutchinson, MN, USA), other devices generate the light within the probe itself (Equanox, Nonin Medical Inc., Plymouth, MN, USA). In both cases, the probe is usually attached to the skin above the tissue of interest. Respective stickers of the probes serve to stabilize the probe’s position over longer periods, but also restrict entrance of ambient light into the measurement photon pathway (Fig. 2). Transcutaneous NIRS is noninvasive and the applied light intensities are not harmful to the tissue, not causing skin burns even if applied for a longer period.

On its way through the tissue, the applied beam interacts with multiple tissue components. Light is partly reflected at optical surfaces, partly redirected and scattered at certain tissue components, such as mitochondria, with much signal loss during tissue transition. More importantly for NIRS, the light will partly be absorbed when contacting optical pigments, like melanin, myoglobin or hemoglobin. In the latter two cases, the resultant spectrum will change, depending on the oxygenation status of the pigments. One or more detectors are integrated into the NIRS probe to collect a fraction of light eventually returning to the tissue surface. In systems designed to measure at different tissue depth, a composite sticker probe may comprise two or more single detectors. In this case, the measurement depth in tissue depends on the light source-to-detector distance, usually in the range of 1 to several centimeters, under the assumption of a banana- or boomerang shaped pathway of the light from the superficial emitter through the tissue of interest and back to a superficial detector.

In recent years, NIRS has developed from a merely experimental tool to a monitoring tool with broad potential clinical use. NIRS monitors have become smaller, cheaper, rugged, and in addition, the ease of use has greatly improved, e.g., by improved user interfaces and minimizing calibration procedures. NIRS monitors are now available from a number of various companies, including Nonin Medical Inc. (Plymouth, MN, USA), Hutchinson Technology Inc. (BioMeasurement Division, Hutchinson, MN, USA), Covidien (Dublin, Ireland) and others. Whereas some medical companies focus on cerebral oxygenation (e.g., for cardiac surgery or carotid surgery), other companies provide monitors and probes specially designed for peripheral measurement of tissue oxygenation. For neonates, specialized mini-probes are also available. Another interesting development is the further minituriarisation of the devices, now reaching almost pocket size format with the introduction of the SpotCheck monitor (Hutchinson).

## NIRS measurement sites

The most common application of NIRS is the assessment of cerebral oxygenation, e.g. during cardiac surgery (see below). Since near infrared light easily penetrates the skull, it is possible to perform real-time assessment of regional (frontal) cortical oxygenation (rSO_2_) using stickers placed on the patient’s forehead. This region contains blood in a so-called watershed area, i.e. the area that lies between those receiving blood from the anterior and medial cerebral artery. Brain tissue oxygen haemoglobin saturation thus obtained derives mostly from the grey matter in the cerebral cortex and reflects the balance between oxygen delivery and utilisation of that region.

For the measurement of peripheral tissue oxygenation multiple potential measurement sites are available, however only few are established. The measurement sites are usually defined by the underlying musculature under the assumption that the underlying muscle body serves as relatively homogenous tissue compartment. Traditionally, the thenar eminence has been promoted as measurement site for peripheral oxygenation measurement. The thenar eminence is reported to have a relatively thin fat layer, compared to other skin regions, with in addition only minor inter-individual variation. In addition, the thenar eminence is reported to participate in systemic edema formation to a lesser extent than other skin regions. Both factors should result in a more homogenous (muscle-) measurement compartment for NIRS. Another reason is that even in negroids the thenar is almost non-pigmented and thus methodological problems with pigment interference can be circumvented. Modern NIRS technologies appear to eliminate the pigmentation related misreading, making this argument probably less important.

Major clinical disadvantages of the thenar eminence as measurement site are related to problems to fixate the probe on the somewhat conical thenar eminence. Although this problem has been addressed by the medical industry by developing probes that are relatively stable when attached to the thenar (e.g., the Hutchinson InSpectra probe), this issue remains nonetheless relevant when using flat sticker probes that tend to return from a U-shape when moulded to the rounded thenar eminence to the original flat shape (Covidien, Nonin). Eventually this leads to entrance of ambient light into the emitter-tissue-detector pathway, with possibility of artefact reading or total loss of signal.

Physiologically the forearm (not only the thenar) is a predominant site of (reflex-) vasoconstriction in case of circulatory distress. Here, vascular response may occur earlier and more intense than in other regions of the body, making the forearm a suitable site for peripheral NIRS measurement. [5, 6] Furthermore, placement of NIRS sensors on the forearm enables to measure the oxygenation in isolated upper extremity muscle compartments [7]. In our own clinical and prehospital experience, attaching a NIRS probe parallel to the proximal forearm, above the antebrachial muscle, results in a stable probe position also with standard ‘flat’ sticker probes and under ‘real-life’ conditions. Herein, the probe does not require bending and the probe is positioned stable so that passive and active patient movements do not disturb the measurement (Fig. 2). Moreover, supporting our selection of the forearm as measurement site, others have also demonstrated that forearm NIRS is more sensitive than thenar NIRS, independent of measurement depth, to detect circulatory distress, e.g., in experimental hypovolemia [8]. A potential physiological explanation for this difference between thenar and forearm in sensitivity to hypovolemia is that perfusion of the hand is relatively well-preserved during certain cardiovascular challenges. One example is the cold-induced vasodilation during severe hypothermia, which opens the (micro)circulation to the hands in order to prevent cold-induced tissue damage.

An example from our group is presented in Fig. 2.

Alternative measurement sites beside thenar and forearm for peripheral oxygenation NIRS have been suggested by some, but are not established as of yet. They include pectoral and deltoid muscle [9, 10], as well as paravertebral region and vastus lateralis muscle [10]. Physiological disadvantage of these more central measurement sites may be that they are not participating as much in the centralization process and thus may be less sensitive as NIRS sentinel sites for the detection of hypovolemia. However, given the superficial location of organs like the kidney or the intestines in neonates and infants (i.e., by the thin skin and thin fat layer), the transcutaneous NIRS measurement of kidney or gut oxygenation is principally feasible in this group of patients [10–14]. These tempting indications are no standard applications yet and important question of application (sensor geometry, catchment volume) still need to be evaluated.

Primarily, the current NIRS monitors are used to measure tissue oxygenation. In addition some have the option to also estimate the hemoglobin (and myoglobin) concentration in the sample volume (e.g., Nonin). If this will allow to target transfusion therapy remains to be shown.

### Limitations of NIRS

Although NIRS monitoring to assess tissue oxygenation has been greatly improved in recent years, the technique is still hampered with a number of limitations. Obviously, when dealing with chromatic biomolecules, the question raises, to what extent other than the molecules of interest (e.g., hemoglobin) contribute to the NIRS readings. Herein, for example, skin pigmentation may play a role. Another point of debate is the contribution of myoglobin in the NIRS measurement. Hemoglobin and myoglobin share key properties, including similar optical properties. This is less an issue when measuring trans-cranial cerebral oxygenation, since in case of frontal readings only minimal muscle and thus myoglobin is within the photon pathway. However, when addressing peripheral oxygenation, e.g., at the thenar or forearm, then myoglobin may become a major contributor to the overall reading. Since oxygen affinity of myoglobin is markedly stronger compared to hemoglobin (markedly leftshifted P_50_), myoglobin will have a relatively high oxygen saturation even in case of tissue hypoperfusion and hypoxygenation [15]. Thus, if a clear separation of the myo- versus hemo-globin signal is not defined, i.e., myoglobin is mistaken for hemoglobin, then hemoglobin saturation will be overestimated.

Finally, when applying single-use patient sensors, NIRS monitoring is relatively expensive. Since a thorough cost/benefit analysis has not been done for the majority of possible clinical applications, further studies are also required in that respect.

### Current developments

Traditional NIRS technology was restricted to track changes over time, which might be sufficient if a ‘normal’ baseline is assumed and the development of the patient status is monitored from that point on. However, in settings where the state of tissue oxygenation may be compromised at first measurement, e.g., in the shock room or in the battlefield, the collection of absolute readings is highly desired. To end this, current technical developments are aiming to provide absolute measurements both by improved hardware and software, although this remains hampered with principal problems of NIRS measurements in the relatively poorly defined anatomical catchment volume.

### NIRS in aero-medical services

NIRS may be a valuable addition in prehospital care, including the transport of critically ill patients. Special challenges during patient transport occur in aero-medical services by helicopter or airplane. However, surprisingly little information is available on the use of NIRS in aero-medical environments, particularly on the use of peripheral NIRS measurements [16]. Thus, we studied the implementation of NIRS monitoring in a helicopter-based emergency medical system [17]. Summarizing our data, NIRS in this setting appeared feasible, safe (no effect on avionics), and we did not observe a systematic effect of the helicopter environment on the measurement, e.g., by engine start-up or shutdown or cabin vibrations [18]. Further studies will have to demonstrate, how NIRS monitoring can improve patient safety, therapy and ultimately outcome in these demanding transport settings.

## NIRS in Shock room and Trauma

NIRS monitors may be beneficial in monitoring trauma patients in the field [19, 20] and numerous studies have been performed to study the role of NIRS monitoring in the prehospital civil setting and in the prehospital military area [21, 22]. NIRS may be used to detect and guide therapy in states of regional tissue hypoperfusion, even when systemic markers (e.g., blood pressure) are still within the normal range. The (patho-)physiological base of this discrepancy is that peripheral perfusion may be early compromised in states of hypovolemia and other forms of systemic distress, when blood volume and perfusion are redistributed towards the central compartment to protect the so-called vital organs, e.g., the heart and the brain. In this cascade of circulatory centralization, the peripheral musculature is a site early compromised in perfusion and thus oxygenation. Thus, onset of NIRS-detected tissue hypoxygenation partly reflects the vasoconstrictive ability of the body, aiming at maintenance of central, vital organ directed perfusion and blood pressure. In this context it should be stressed that induction of anaesthesia, as a frequent step in the treatment cascade of emergency (trauma) patients per se may affect tissue oxygenation. Changes in NIRS reading could thus result either from changes in the underlying pathology (e.g., ongoing blood loss) or from the induction of anesthesia (e.g., peripheral anesthesia-induced vasodilation). Therefore, one should know the impact of general anesthesia per se on NIRS measurements. However, the impact of (an induction of) anesthesia itself on NIRS derived measurements remained controversial [23].

In a prospective, nonrandomized, observational, descriptive study, normal human volunteers (n = 707) and patients admitted to the resuscitation area of a Level I trauma center (n = 150) were included to establish the normal ranges of thenar saturation (StO_2_) using the InSpectra tissue spectrometer. The thenar StO_2_ values clearly discriminated the normals or no shock patients and the patients with severe shock [1]. In this study, thenar StO_2_ values were 87 ± 6 % for healthy volunteers, 83 ± 10 % for non-shock patients; 83 ± 10 % for mild shock patients, 80 ± 12 % for moderate shock patients; and 45 ± 26 % for severe shock patients [1]. If these StO_2_-values resembling this categorization also hold true for other NIRS devices, requires further research.

NIRS monitoring may also help to differentiate at an early stage between survivors and nonsurvivors with every 10 % decrease in thenar saturation (StO_2_) increasing mortality threefold [20]. Finally, it has been shown that if a patient is able to maintain StO_2_ above 75 %, there is a high probability of not developing organ dysfunction and death after severe trauma [24, 25].

## NIRS in cardiac surgery

As with peripheral tissues, low regional (frontal) cortical oxygenation (rSO_2_) levels provide an indication of a mismatch between cerebral perfusion or oxygen delivery, and regional oxygen requirements. rSO_2_ has been shown to correlate well with jugular venous bulb saturation, which is the standard for assessing global cerebral saturation [26]. The typical range of rSO_2_ is 55–80 % and absolute rSO_2_-values <50 % or a 20 % drop from individual rSO_2_ baseline are commonly considered as intervention trigger. The incidence of these findings in patients undergoing coronary artery bypass grafting (CABG) is as high as 42 % [27]. An even higher incidence has been reported for pediatric cardiac surgery [28]. Moreover, a rSO_2_ <45 % or a 25 % drop from individual baseline values are considered a critical threshold for unfavourable neurological outcome. For instance, a study involving 100 cardiac surgical patients showed that significantly more impairments in postoperative cognitive function occurred in those patients with either a nadir rSO_2_ below 35 % or with rSO_2_-values below 40 % for more than 10 min [29]. Similarly, patients undergoing aortic arch surgery who spent more than 30 min under the absolute rSO_2_ threshold of 60 % had an extended hospital stay of 4 days leading to substantial additional costs [30].

This technique has been extensively used in patients undergoing cardiac surgery in order to find an association between measurements of cerebral oxygenation and postoperative outcome. Several studies have found an association between intraoperative cerebral oxygen desaturation and postoperative cognitive dysfunction, stroke, and prolonged hospital stay. In a landmark study, 200 CABG patients were randomized to receive either blinded rSO_2_-monitoring or an intervention algorithm based on rSO_2_ readings. In the intervention group there were significantly less major complications (death, stroke, renal and respiratory failure) and a shorter length of stay (LOS) in the intensive care unit [31]. Similar reductions in major complications have been found in a before/after treatment protocol implementation based on rSO_2_ in more than 2000 cardiac surgical patients [32]. Furthermore, there was an inverse relationship between mean intraoperative rSO_2_ and a hospital stay >10 days, and low rSO_2_ was associated with “outlier” patients, i.e. those having the longest recovery times [31]. Similarly, the occurrence of rSO_2_ values below 50 % increased the risk of cognitive dysfunction and prolonged hospital stay threefold [33], particularly in elderly patients [33, 34]. A cost-effective analysis based on these data revealed that savings due to avoidance of cerebrovascular accidents and reduction in LOS by far (factor 5) exceeded the additional costs of rSO_2_ monitoring [35].

However, some limitations should be kept in mind that might interfere with these findings. The first relate to patient related factors such as advanced age and comorbidities, which are common in this patient population. As a consequence, baseline rSO_2_-values of cardiac surgery patients were lower compared to healthy volunteers. Second, the procedural complexity of cardiovascular surgery and the equipment frequently involved (e.g. cardiopulmonary bypass) can affect neurological outcome by itself. The incidence of stroke, for instance, ranges from 1 to 3 % in coronary artery bypass grafting [36, 37] and may be substantially higher in aortic surgery. Of note, nearly 75 % of all strokes occurred among the 90 % of patients at low or medium preoperative risk, suggesting that many of these strokes may be preventable by adequate monitoring [36]. In addition, patients who had perioperative stroke were at a significantly increased risk for death. A study on 35,733 consecutive patients undergoing isolated CABG surgery from 1992 to 2001 showed that survival at each time point was lowest among patients who had *hypoperfusion* strokes compared to those with embolic of no strokes [37]. A study based on 503,478 records from the Society for Thoracic Surgeons (STS) database calculated a cumulative “Major Organ Morbidity or Mortality score” consisting of death within 30 days, renal failure requiring dialysis, permanent stroke, need for re-operation for any reason, >48 h ventilation, and mediastinitis/deep sternal infection. The incidence of this composite endpoint was 13.4 % [38]. Further postoperative problems include delirium (incidence 10-60 %) and postoperative cognitive dysfunction (incidence 24–53 %) that lead to a longer hospital length of stay.

A recent study suggests that NIRS can also be used to assess cerebral autoregulation [39]. Another study suggests that the choice of anaesthetic may also be important: a sevoflurane-based anaesthesia was associated with better short-term postoperative cognitive performance than propofol [40]. Even preoperative baseline cerebral oxygenation appears to be predictive of short- and long-term morbidity and mortality [41]. Of note, cerebral desaturation occurs not only in cardiac procedures performed using cardiopulmonary bypass (on-pump), but also during off-pump CABG, particularly when the heart is tilted for distal anastomoses at the lateral and posterior walls [42].

Tissue oxygenation has also been measured at the thenar site in patients undergoing cardiac surgery with cardiopulmonary bypass (CPB). With initiation of CPB, StO_2_ declined by 13 % with a delayed increase in lactate and base deficit. Importantly, the minimum StO_2_ value preceded the maximum lactate level by an average time of 94 min, suggesting that NIRS identifies perfusion deficits much earlier than conventional metabolic markers [43].

In conclusion, peri-operative assessment of cerebral oxygenation using NIRS provides useful real-time information about the adequacy of cerebral perfusion/oxygenation as well as the incidence and time course of cerebral hypoxia, and may help to identify the causes and to find methods of preventing and managing cerebral hypoperfusion and hypoxia during cardiac surgery. In addition, NIRS monitoring of cerebral oxygenation is not temperature or pulse dependent and immediately reflects patient reactions or efficacy of therapeutic interventions.

## NIRS in non-cardiac surgery

Up to now little information exists on the value of cerebral NIRS monitoring during non-cardiac surgery. One study randomized 122 elderly patients undergoing major abdominal surgery to a control group or an intervention group in which rSO_2_ was kept ≥75 % of individual baseline values. As a result, less cognitive decline and shorter hospital stay was observed in the group where cerebral hypoxia was prevented [44].

Several studies looked at the role of cerebral NIRS monitoring in patients undergoing carotid endarterectomy. For instance, cerebral rSO_2_ monitoring was compared to transcranial doppler and awake testing under local anesthesia; transcranial doppler was less accurate than NIRS in predicting the need for carotid shunting [45, 46]. In another study, transcranial doppler, NIRS, and stump pressure measurement provided similar accuracy for the detection of cerebral ischemia during carotid surgery, while lower accuracy was found for SEP monitoring. Because of the high rate of technical difficulties (21 %), transcranial doppler monitoring was the least practical of the investigated monitoring devices [47]. A much bigger study involving 954 patients with >70 % carotid stenosis incl. 39 % with contralateral occlusion as confirmed by doppler and angiography addressed the question which change in rSO_2_ needs shunting. The results suggested that a relative decrease in rSO_2_ was a better indicator than an absolute decrease, that a decrease ≤20 % was no indication for a shunt, and that a decrease >20 % not always predicted neurological complications [48]. The authors concluded that a short decrease >20 % may be tolerated and 2 min should be allowed for autoregulatory fluctuations. Furthermore, the study found no impact of any pharmaceutical interventions on NIRS (unlike on SEPs and EEG) [48]. Finally, no correlation was found between rSO_2_ and blood pressure values during carotid cross-clamping suggesting that an excessive rise of blood pressure is not necessary to guarantee adequate cerebral blood perfusion [49].

Another application of cerebral NIRS monitoring is during shoulder surgery in the beach-chair position. In these operations, cerebral desaturation is provoked either by postural hypotension, by head and neck manipulation leading to changes in cerebral blood flow, or by thromboembolic events. Compared to the lateral decubitus position, patients undergoing shoulder arthroscopy in the beach chair position suffered significantly more cerebral desaturations (80 vs. 0 %) despite similar baseline rSO_2_ values [50]. Consequently, patients suffering from cerebral desaturation had higher rates of postoperative nausea (50 vs. 7 %) and vomiting (27 vs. 3 %), whereas no neurologic deficits were observed, likely because of the limited duration of the surgical procedure [50].

Thoracic surgery with one-lung ventilation seemed to be associated with a significant decrease in rSO_2_ [51], and minimal rSO_2_-values correlated positively with postoperative complications [52].

## Conclusion

NIRS offers non-invasive online monitoring of tissue oxygenation in a wide range of clinical scenarios. A common application is to measure cerebral oxygenation (rSO_2_), e.g. during cardiac surgery. Tissue hypoxia occurs frequently in the perioperative setting, particularly in cardiac surgery. Therefore, measuring and obtaining adequate tissue oxygenation may prevent (postoperative) complications and may thus be cost-effective. NIRS monitoring may also be used to detect tissue hypoxia in (prehospital) emergency settings, where it has prognostic significance and enables monitoring of therapeutic interventions. Optimal therapeutic agents and strategies for augmenting tissue oxygenation have yet to be determined.
